# Rhomboid-Like-2 Intramembrane Protease Mediates Metalloprotease-Independent Regulation of Cadherins

**DOI:** 10.3390/ijms20235958

**Published:** 2019-11-27

**Authors:** Chiara Battistini, Michael Rehman, Marco Avolio, Alessia Arduin, Donatella Valdembri, Guido Serini, Luca Tamagnone

**Affiliations:** 1 Department of Oncology, University of Torino School of Medicine, 10060 Candiolo, Torino, Italy; chiara.battistini@ircc.it (C.B.); marco.avolio@ircc.it (M.A.); donatella.valdembri@ircc.it (D.V.); guido.serini@ircc.it (G.S.); 2 Candiolo Cancer Institute, FPO-IRCCS, 10060 Candiolo, Torino, Italy; michael.rehman@outlook.com (M.R.); alessia2323@gmail.com (A.A.); 3 Istituto di Istologia ed Embriologia, Università Cattolica del Sacro Cuore, 00168 Rome, Italy; 4 Fondazione Policlinico Universitario “A. Gemelli”, IRCCS, 00168 Rome, Italy

**Keywords:** rhomboid, E-cadherin, proteolytic cleavage, cell migration, TNFα

## Abstract

Cadherins are a major family of cell–cell adhesive receptors, which are implicated in development, tissue homeostasis, and cancer. Here, we show a novel mechanism of post-translational regulation of E-cadherin in cancer cells by an intramembrane protease of the Rhomboid family, RHBDL2, which leads to the shedding of E-cadherin extracellular domain. In addition, our data indicate that RHBDL2 mediates a similar activity on VE-cadherin, which is selectively expressed by endothelial cells. We show that RHBDL2 promotes cell migration, which is consistent with its ability to interfere with the functional role of cadherins as negative regulators of motility; moreover, the two players appear to lie in the same functional pathway. Importantly, we show that RHBDL2 expression is induced by the inflammatory chemokine TNFα. The E-cadherin extracellular domain is known to be released by metalloproteases (MMPs); however, here, we provide evidence of a novel MMP-independent, TNFα inducible, E-cadherin processing mechanism that is mediated by RHBDL2. Thus, the intramembrane protease RHBDL2 is a novel regulator of cadherins promoting cell motility.

## 1. Introduction

Cell regulation is largely due to molecules that are localized at the plasma membrane. They mostly include receptors for extracellular signals, transporters, and adhesive proteins. Cadherins represent one of the most important families of cell–cell adhesive proteins, playing a fundamental role in development, tissue homeostasis, and cancer [1,2,3,4]. Interestingly, E-cadherin, which is mainly expressed in epithelial cells, is known to have additional functions beyond establishing stable cell–cell contacts, by controlling cell signaling cascades driving cell differentiation, proliferation/self-renewal, and cell migration [5,6]. In particular, the role of E-cadherin in controlling cell migration is incompletely understood. For instance, its surface expression appears to also be relevant in carcinoma cells that are not engaged in cell-cell contacts, which suggests that its function goes beyond stabilizing the quiescent epithelial cell layers [7,8,9].

Notably, the function of cell surface proteins, including cadherins, is often controlled by post-translational mechanisms, such as internalization or proteolytic processing. Intramembrane proteases are a relatively less investigated family of proteolytic enzymes, which are embedded in the lipid bilayer, where they can cleave the transmembrane domain of associated proteins, leading to the release of the extracellular portions [10,11]. The Rhomboid family of multispanning intramembrane serine proteases is expressed in all phyla [12]. The first identified rhomboid gene was *rhomboid-1*, which activates EGFR signaling in *Drosophila melanogaster* by releasing its cognate ligands from their membrane-tethered precursors [13,14]. In mammals, five different rhomboid-like proteases have been described, i.e., RHBDL1-4 and the mitochondrial presenilin-associated rhomboid-like protease (PARL), and their functional role in development and human disease is under investigation [15,16,17]. No substrates have yet been identified for RHBDL1 and 3 [15,18]. RHBDL4 is localized in the Endoplasmic Reticulum, where it is involved in ER-associated degradation and also potentially in oncogenic signaling, but its substrates are still uncertain [19,20,21]. A few diverse candidate substrates have been identified for mammalian RHBDL2, such as ephrin-B3, EGF, Thrombomodulin, and the paralog protein CLEC14A [17,22,23,24,25,26], but its functional role remains still elusive; notably, these substrates are also known to be shed by metalloproteases, like ADAMs [27,28,29]. The general mechanism of rhomboid-mediated catalysis is thought to be similar to that of other serine proteases: in particular, RHBDL2 presents a catalytic dyad that is formed by a serine and a histidine located in the fourth and the sixth transmembrane domains, respectively [30]. Due to the transmembrane localization of these enzymes, their hydrophilic catalytic site remains in a closed conformation in the absence of substrates [31]. In fact, the transmembrane substrates of rhomboid proteases are characterized by the presence of helix-destabilizing residues that enter the active site, thanks to a broad conformational rearrangement of the protease (gate opening) that is induced by the substrate via the allosteric regulatory domains [32].

In this study, we report for the first time that the extracellular domain of E-cadherin is shed by RHBDL2 protease expressed in tumor (and non-tumor) cells. Moreover, while E-cadherin cleavage is known to also be mediated by metalloproteases (MMPs), here we show an MMP-independent mechanism of intramembrane processing of cell surface E-cadherin, which is mediated by RHBDL2. We found that RHBDL2 is also capable of cleaving the homologous endothelial cell specific VE-cadherin. Interestingly, we discovered that RHBDL2 expression is specifically induced in tumor cells by the inflammatory signal TNFα, which leads to E-cadherin cleavage and shedding. In addition, our data suggest that RHBDL2 activity controls cancer cell migration by E-cadherin functional inactivation.

## 2. Results

### 2.1. RHBDL2 Controls Cancer Cell Migration

**Figure 1:**
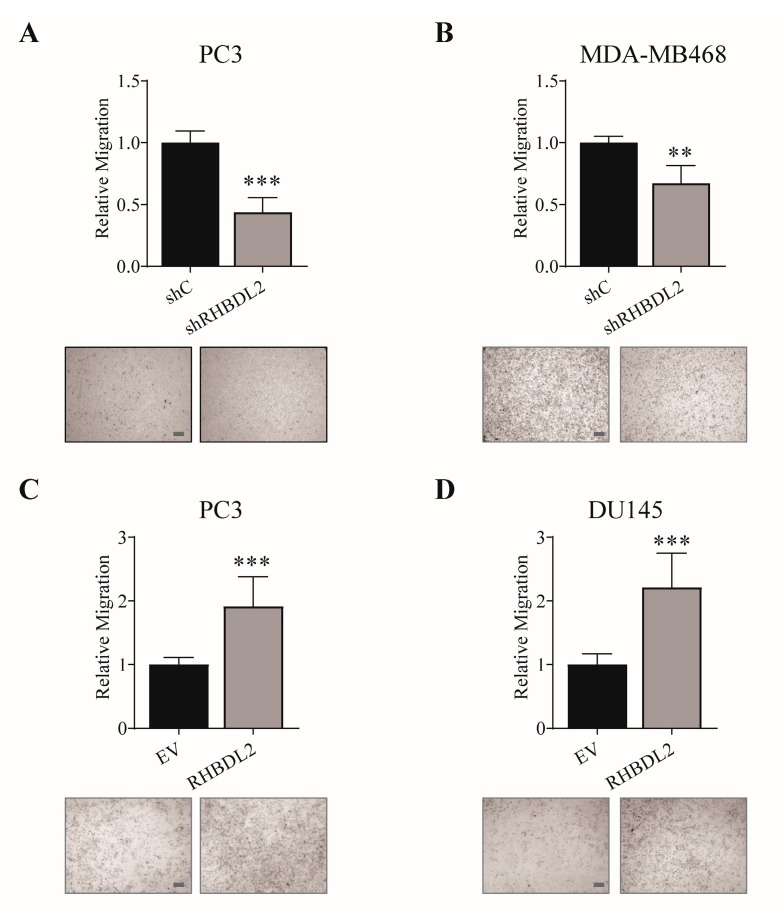
RHBDL2 controls cancer cell migration. The migration of PC3 (**A**) and MDA-MB468 (**B**) cells, either control (shC) or silenced for RHBDL2 (shRHBDL2), was assessed using Transwell chamber inserts. Similarly, it was quantified the migration of PC3 (**C**) and DU145 (**D**) cells stably transduced with either a RHBDL2-expressing construct, or an empty vector (EV). A representative field of the inserts containing migrated cells, stained with crystal violet, is shown below each graph; scale bars: 500 μm. Data are the mean ± SD from three independent experiments. Statistical significance: ** *p* < 0.005; *** *p* < 0.0005.

In a high throughput functional screening in PC3 prostate carcinoma cells, the knock-down of intramembrane protease gene RHBDL2 was serendipitously found to inhibit cell migration. This was consistent with data that were shown in a previous study on normal keratinocytes [24]; however, the potential relevance of this protease in cancer cell migration had not been formerly investigated. Thus, we decided to focus on RHBDL2 by performing new independent gene silencing experiments in the PC3 cells, and confirming its functional relevance in another invasive cancer cell line, the triple-negative breast carcinoma cells MDA-MB468 characterized by a high expression of the protease (Figure S1A,B and Figure 1A,B). Notably, RHBDL2 knock-down did not cause significant changes in cell morphology, viability, or growth rate, despite the visible impact on cell migration (Figure S1C,D).

By a complementary approach, we overexpressed RHBDL2 in PC3 cells, as well as in another prostate carcinoma model, DU145, characterized by low endogenous levels of the protease (Figure S1A). The upregulation of RHBDL2 caused a significant induction of cell migration (Figure 1C,D). Of note, also in this case, the cell morphology or growth rates were not affected (Figure S1E,G), which validates our conclusion that RHBDL2 controls a mechanism that specifically regulates cancer cell migration.

### 2.2. RHBDL2 Modulates E-Cadherin Expression and Shedding in Human Cancer Cells

We postulated that the observed increase in cancer cell migration could be explained by the enrichment in a motogenic factor shed from the cell surface, or alternatively by the post-translational modification of a transmembrane protein basally restraining cell migration when considering the intramembrane protease activity of RHBDL2. Intriguingly, we extensively tested, in vain, the ability of the conditioned medium of RHBDL2 overexpressing cells to trigger cancer cell migration (Figure S2A,B, and data not shown). Moreover, while considering its reported activity in EGF shedding [23], we specifically analyzed EGFR activation in cells overexpressing RHBDL2, but could not confirm this mechanism in our cancer cell models (Figure S2C). Another cell surface protein that is found to be cleaved by RHBDL2 in (non-tumor) epithelial cells is thrombomodulin [24,25]. This transmembrane protein substrate rather acts as an inhibitor of cell migration, whose function is thought to be inactivated by RHBDL2. However, thrombomodulin expression was almost undetectable in our cancer cell models, and we did not find its released extracellular portion upon RHBDL2 upregulation (Figure S2D).

**Figure 2:**
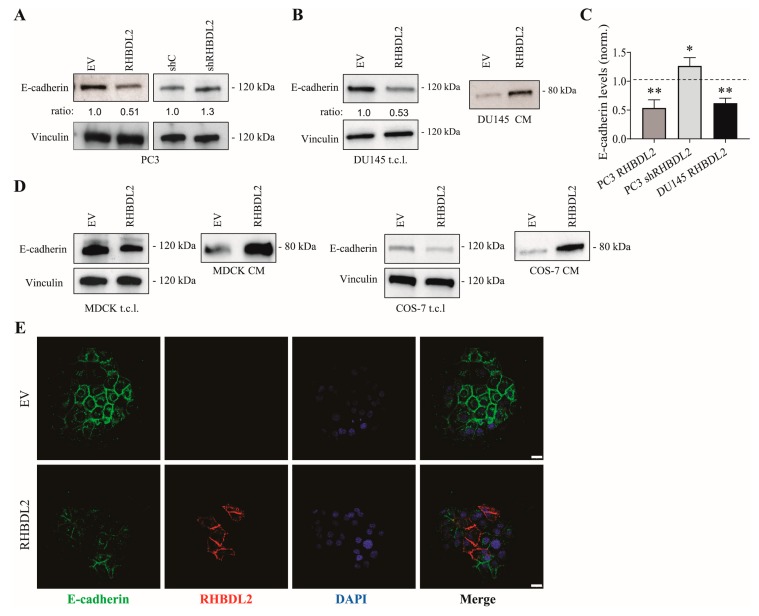
RHBDL2 downregulates E-cadherin levels in prostate cancer and non-tumoral cells, by proteolytic cleavage and E-cadherin shedding. (**A**) Western Blotting (WB) analysis showing the impact of RHBDL2 overexpression (left lanes) or knock-down (right lanes) on E-cadherin levels, in total lysates of PC3 cells. E-cadherin levels (relative to vinculin, providing a loading control) were quantified and normalized to respective control conditions (set to 1). Different independent batches of transduced cells were analyzed, yielding consistent results (see panel C). (**B**) WB analysis of E-cadherin in total lysates (t.c.l.) and conditioned media (CM) of DU145, stably transduced with a RHBDL2-expressing construct or an empty vector (EV). Upon RHBDL2 overexpression, E-cadherin levels decreased in total lysates while the shedding of its soluble extracellular domain increased. E-cadherin levels in t.c.l. (relative to vinculin, providing a loading control) were quantified and normalized to respective control conditions (set to 1). Different independent batches of transduced cells were analyzed, yielding consistent results (see next panel). (**C**) The plot shows E-cadherin protein level variations (normalized to respective controls, dashed line) in three independent batches of PC3 or DU145 cells subjected to RHBDL2 genetic manipulation, and analyzed by WB as shown in panels A-B. Statistical significance: * *p* < 0.05; ** *p* < 0.005. (**D**) Western Blotting analysis of E-cadherin in total lysates (t.c.l.) and conditioned media (CM) of two non-tumoral kidney-derived cell lines, MDCK and COS-7, stably transduced with a RHBDL2-expressing construct or an empty vector (EV). Vinculin staining provided a loading control. (**E**) Immunofluorescence analysis of E-cadherin (green) and RHBDL2 expression (red) in DU145 cells that were transduced with a RHBDL2-expressing construct or an empty vector (EV); the nuclei were stained by DAPI (blue). Scale bars: 25 µm.

Thus, we considered the possibility that RHBDL2 might have additional unexpected substrates in cancer cells. A list of putative RHBDL2 targets, which were identified by quantitative proteomic screening, was recently reported, and the cell surface N-Cadherin molecule was among the top hits [33]. In this study, the authors could not experimentally confirm a role of RHBDL2 in N-Cadherin cleavage, which is also consistent with our data (Figure S2E). However, we thought to expand our analysis to include additional members of the cadherins family, which may share structural features beyond the primary sequence. In particular, we observed that the E-cadherin protein levels markedly decreased in PC3 cancer cells upon RHBDL2 overexpression, while they conversely increased upon silencing the protease (Figure 2A,C); notably, these changes were independent of transcriptional regulation (Figure S3A). RHBDL2 in DU145 carcinoma cells indued similar effects, which express higher basal levels of E-cadherin (Figure 2B,C, and Figure S3B); thus, we could also detect a striking increase in the shedding of soluble E-cadherin extracellular domain in the conditioned medium of these cells, in response to RHBDL2 expression (Figure 2B). Interestingly, RHBDL2 expression also induced E-cadherin cleavage in non-tumoral cells: in fact, we observed decreased cell-associated E-cadherin, and increased ectodomain shedding, upon RHBDL2 transfection, in kidney-derived MDCK and COS-7 cell lines (Figure 2D).

We observed that, in DU145 cells (bearing bona fide epithelial features), E-cadherin is extensively localized in adhesive contacts at the cell surface (Figure S3C) by immunostaining analysis of non-permeabilized cells; in contrast, E-cadherin is not engaged in cell-cell contacts in PC3 cells (Figure S3D), having rather mesenchymal morphology (see Figure S1E). In keeping with Western blotting data, RHBDL2 protease greatly reduced cell surface E-cadherin in both cancer cell models (Figure S3C,D), although this was expectedly more evident in DU145. Moreover, in a separate experiment, we double-stained these cells to detect both RHBDL2 and E-cadherin, which allowed for appreciating that the cells with highest expression of the protease are also prominently depleted of E-cadherin (Figure 2E).

**Figure 3:**
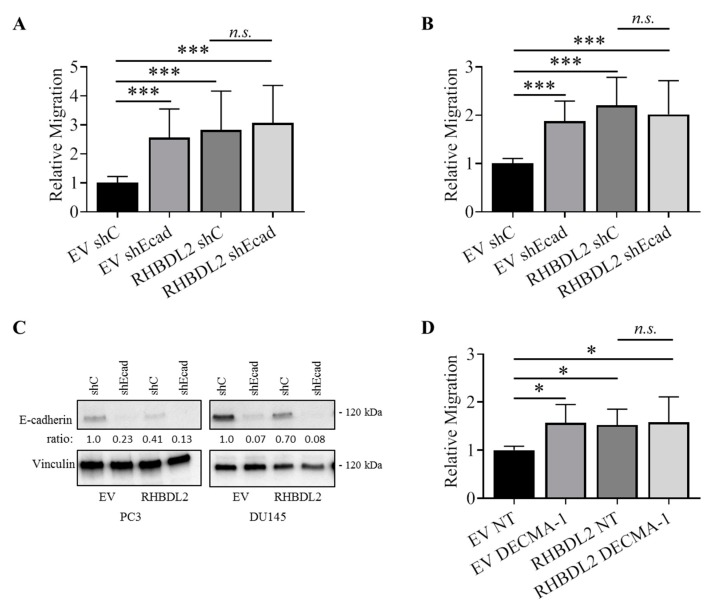
RHBDL2 and E-cadherin act in the same functional pathway regulating cancer cell migration. PC3 and DU145 cells were first stably transduced with an empty vector (EV) or a RHBDL2-expressing construct; then each cell line was further transduced with a scramble shRNA (shC) or a shRNA specifically targeting E-cadherin (shEcad). (**A**,**B**) The migration of these genetically manipulated PC3 (**A**) and DU145 (**B**) cells was analyzed in Transwell assays (as shown in Figure 1). (**C**) The efficiency of E-cadherin silencing in the cells above was analyzed by Western Blotting. (**D**) Wound healing assays performed with DU145 cells, control (EV) and overexpressing RHBDL2, in presence or absence of the E-cadherin function-blocking antibody DECMA-1 (20 µg/mL); the graph shows the quantification of relative cell migration in the wound, normalized to controls. Data are the mean ± SD from three independent experiments. Statistical significance: * *p* < 0.05; *** *p* < 0.0005.

We performed cell migration experiments in which E-cadherin was independently targeted by gene knock down with shRNA, or by means of RHBDL2 overexpression in order to tackle the role of RHBDL2 in E-cadherin functional interference; in either case, we similarly observed a cell migration increase in association with E-cadherin downregulation, and this was confirmed in two distinct cancer cell models (Figure 3A–C). Importantly, when RHBDL2 overexpression was imposed in cells that were depleted of E-cadherin, we did not observe any additional impact in the regulation of cell migration (Figure 3A,B), which suggested that the two proteins act in the same functional pathway. Interestingly, we obtained the same results in the wound healing assays of DU145 cells treated with the E-cadherin function-blocking antibody DECMA-1: thus, both E-cadherin functional interference and RHBDL2 overexpression increased DU145 cell migration, but the two mechanisms were not additive (Figure 3D). Notably, we did not detect any change in the morphology or proliferation rate of our cellular models upon *CDH1* gene silencing (Figure S4A–C), which is consistent with that observed in association with RHBDL2-induced E-cadherin cleavage in the same cells (Figure S1E–G).

We treated RHBDL2-overexpressing (and control) DU145 cells with the serine protease inhibitor 3,4-dichloroisocoumarin (DCI), which is capable of hindering RHBDL2 activity, in order to validate the functional relevance of RHBDL2 enzymatic activity in E-cadherin processing and cell migration [24]; indeed, the treatment with DCI decreased RHBDL2-induced E-cadherin shedding, and it significantly reduced the gain in cell migration that was induced by RHBDL2 (Figure S4D,E). Taken together, these results strongly suggest that RHBDL2-driven proteolytic regulation of E-cadherin is a novel mechanism that is responsible for cell migration enhancement.

### 2.3. RHBDL2 Interacts with E-Cadherin and VE-Cadherin, and Catalyzes Their Cleavage and Extracellular Shedding

**Figure 4:**
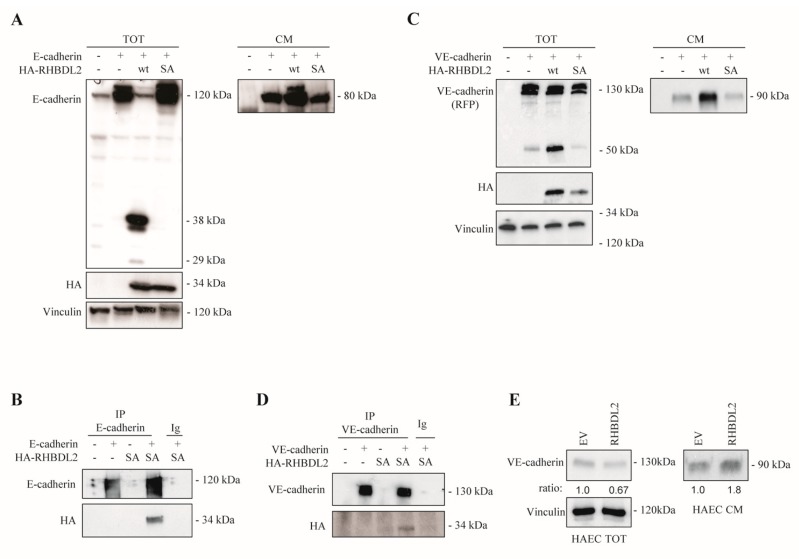
RHBDL2 interacts with E-cadherin and VE-cadherin, catalyzing their proteolytic cleavage and extracellular shedding. (**A**) Western Blotting showing total lysates (TOT) and conditioned media (CM) of HEK-293T cells transfected with E-cadherin and HA-tagged RHBDL2, either wild-type (wt) or mutated in the catalytic site, serine to alanine (SA). For total lysates an antibody directed against the cytoplasmic domain of E-cadherin was used, thereby showing the generation of intracellular cleaved fragments upon co-expression with wt RHBDL2, but not with SA mutant. Expression of HA-tagged rhomboids is shown below, and vinculin provided a loading control. CM collected from the same cells were analyzed with an antibody directed again E-cadherin extracellular domain, revealing that co-expression with wt (but not SA) RHBDL2 increased its shedding. (**B**) Lysates from HEK293T cells transfected with E-cadherin and E-cadherin and RHBDL2-SA mutant were immunoprecipitated with an antibody recognizing E-cadherin, or with an unrelated antibody (Ig). Western Blotting against HA-tag revealed co-immunoprecipitation of RHBDL2 with E-cadherin. A reverse immunoprecipitation experiment is shown in Figure S6A. (**C**) Western Blotting showing total lysates (TOT) and conditioned media (CM) of HEK293T cells transfected with VE-cadherin (RFP-tagged at the C-tail) and HA-tagged RHBDL2, wild-type (wt) or catalytic inactive mutant SA (as in panel A). For total lysates an antibody directed against RFP-tag was used, showing the generation of an intracellular cleaved fragment upon co-expression with wt RHBDL2, but not with SA mutant. Expression of HA-tagged rhomboids is shown below, and vinculin provided a loading control. CM collected from the same cells were analyzed with an antibody directed again VE-cadherin extracellular domain, revealing its increased shedding when co-expressed with wt (but not SA) RHBDL2. (**D**) Lysates from the HEK293T cells transfected with VE-cadherin and VE-cadherin and RHBDL2 SA mutant were immunoprecipitated while using an anti-RFP antibody, or an unrelated antibody (Ig). Western Blotting against HA-tag revealed co-immunoprecipitation of RHBDL2 with VE-cadherin. (**E**) Western Blotting analysis of VE-cadherin levels in total lysates (TOT) and conditioned media (CM) of Telo-HAEC cells, stably transduced with a RHBDL2-expressing construct or an empty vector (EV): upon RHBDL2 overexpression, VE-cadherin decreases in total lysates while the shedding of its soluble extracellular domain is increased. Vinculin staining provided a loading control. The experiment was repeated with different independent batches of transduced cells, showing similar results.

We co-expressed E-cadherin in another cell model (HEK-293T non-tumoral cells), together with wild-type RHBDL2 or its enzymatic site-mutated version (SA) lacking protease activity, to confirm the specificity of this regulatory mechanism; this also allowed for analyzing the impact of RHBDL2 catalytic activity on its substrate in more detail. Indeed, we found that the E-cadherin levels dramatically dropped upon RHBDL2 upregulation, while there was no effect upon the overexpression of a catalytic inactive version of the protease (Figure 4A). Moreover, the complex of the enzyme and its substrate could not be isolated, which is consistent with a high dissociation rate of the products of the proteolytic reaction. Instead, we could easily recover a specific complex of E-cadherin in association with catalytic inactive RHBDL2-SA (Figure 4B).

Furthermore, we asked whether this regulatory activity of RHBDL2 could affect another member of the Cadherin family, which is specifically found in endothelial cells, VE-cadherin. Indeed, the association with RHBDL2 and proteolytic targeting of VE-cadherin was similarly validated in both heterotypic and endothelial cell models (Figure 4C–E). Intriguingly, different from cancer cells, RHBDL2 overexpression induced a morphological rearrangement in endothelial cells, which was similar to that observed upon cell-cell adhesion dissociation in low-Ca^2+^ condition (Figure S5A,B); consistently, in response to RHBDL2, endothelial cell migration was also moderately, but significantly, induced (Figure S5C). Thus, our data indicate that RHBDL2 is capable of proteolytically regulating E-cadherin, and the homologous protein VE-cadherin, in different cell types, which putatively inactivates their function on the cell surface.

### 2.4. RHBDL2 Expression Is Induced by the Inflammatory Cytokine TNFα

**Figure 5:**
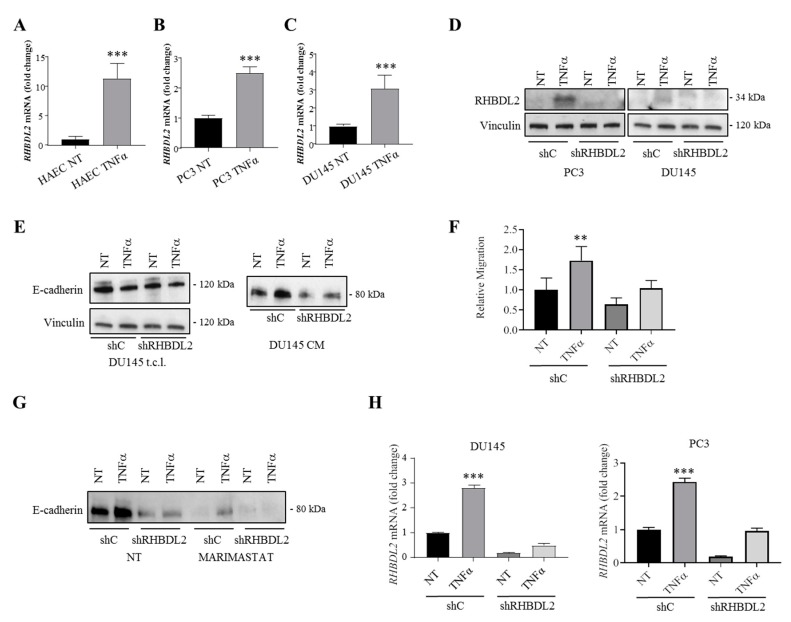
TNFα up-regulates RHBDL2 expression, inducing E-cadherin shedding in a MMP-independent manner. (**A**–**C**) RHBDL2 mRNA levels were quantified by real-time qPCR in Telo-HAEC (**A**), PC3 (**B**) and DU145 (**C**) cells after 6 h of treatment with TNFα 10 ng/mL. Data are the mean ± SD from three independent experiments. Statistical significance: *** *p* < 0.0005. (**D**) TNFα-induced RHBDL2 upregulation in PC3 and DU145 cells was detectable also by Western Blotting, after cell treatment for 24 h with TNFα 10 ng/mL. RHBDL2-silenced cells treated in parallel provided a specificity control for the detected band; vinculin was used as a loading control. (**E**) Western Blotting analysis of total lysates (t.c.l.) and conditioned media (CM) of DU145 control (shC) and RHBDL2-silenced (shRHBDL2) cells, untreated (NT) or TNFα treated. The cells were treated for 6 h with TNFα 10 ng/mL, then medium was replaced with fresh TNFα at the same concentration and CM was collected after overnight incubation. Vinculin staining was used as a loading control. (**F**) PC3 cells were first stably transduced with scramble shRNA (shC) or a shRNA specifically targeting RHBDL2 (shRHBDL2), then treated for 6 h with TNFα 10 ng/mL to induce RHBDL2 expression, and finally allowed to migrate overnight. The panel shows the quantification of migrated cells in Transwell assays. Data are the mean ± SD from three independent experiments. Statistical significance: ** *p* < 0.005. (**G**) Western Blotting analysis of (TNFα-induced) E-cadherin released in conditioned media (CM) harvested from DU145 control (shC) and RHBDL2-silenced (shRHBDL2) cells (same as analyzed in previous panels), in absence or in presence of Marimastat 20 µM. Three independent experiments were performed, showing consistent results. (**H**) RHBDL2 expression was verified by qPCR in DU145 cells analyzed in panel E and G, and in PC3 cells analyzed in (**F**).

It is currently unknown how the RHBDL2 levels are regulated in cancer context. The inflammatory cytokine TNFα is a known inducer of metalloproteases [34,35,36,37]; thus, we asked whether TNFα could also induce RHBDL2 expression. Indeed, we found a remarkable induction of RHBDL2 expression by TNFα in both endothelial and cancer cells (Figure 5A–D). Moreover, this effect was impaired by the inhibitors of NF-κB (Figure S6B), a known TNFα effector in cancer cells [38].

Interestingly, we found that TNFα induced E-cadherin cleavage and extracellular shedding in DU145 cancer cells, which was dependent on the upregulation of endogenous RHBDL2, as demonstrated by the gene knock-down experiments (Figure 5E).

Moreover, TNFα treatment induced PC3 cell migration, but such an effect was blunted upon RHBDL2 silencing, which suggested that this protease might act as downstream effector of the cytokine in the regulation of cancer cell migration (Figure 5F).

### 2.5. RHBDL2 Mediates MMP-Independent E-Cadherin Cleavage in Cancer Cells

While RHBDL2 appeared as the main effector of TNFα-induced E-cadherin cleavage, our data showed that a basal level of E-cadherin shedding was visible and maintained in cancer cells also upon RHBDL2 depletion, being presumably accountable by the activity of diverse MMPs [39,40,41]. It is worth noting that the MMP inhibitors have been repeatedly considered as potential drugs in cancer therapy, due to their broad impact on protein regulation [42]. Thus, we aimed at stripping MMP-activity in DU145 cancer cells by means of the broad inhibitor Marimastat, which is ineffective on intramembrane protease RHBDL2. This treatment strikingly reduced E-cadherin cleavage and shedding in basal conditions, but it did not prevent TNFα induced E-cadherin processing, which was confirmed to be reliant on RHBDL2 and entirely MMP-independent (Figure 5G). This novel mechanism of MMP-independent cleavage of E-cadherin was further confirmed in RHBDL2 overexpressing cells (Figure S6C,D). Notably, MMPs have also been reported to regulate cell migration, due to their activity on extracellular matrix components and cell surface proteins, beyond E-cadherin [43]. Intriguingly, while the migration of certain cancer cells that were reliant on RHBDL2 was essentially independent of MMP activity, for others the migration was conditioned by MMPs, both basally and upon RHBDL2 upregulation (Figure S6E,F).

## 3. Discussion

Surface-exposed proteins are pivotal regulators of cell signaling, and their function is often regulated at the post-translational level by protease-mediated processing. In particular, intramembrane proteases of the rhomboid family are highly conserved in evolution, from invertebrates to humans; they have been mainly investigated in development, while their function in cancer is poorly understood [16,17,44]. A preliminary gene expression analysis of public databases indicated that RHBDL2 expression significantly correlates with poor prognosis in breast cancer, pancreatic adenocarcinoma, clear cell kidney cancer, and low-grade glioma patients (Kmplot.com platform and TCGA PanCancer atlas, see Figure S7); this is consistent with previous data regarding RHBDL2 overexpression in basal-like breast carcinomas as compared to normal breast [45]. However, very little was known about the actual functional impact of RHBDL2 protease in cancer cells. Moreover, RHBDL2 has been previously reported to target a few unrelated substrate proteins, including EGF. Yet, although RHBDL2-dependent EGF activation was proposed to impact cancer cell functions, this mechanism was not further investigated.

Here, we report the finding of a novel relevant substrate of RHBDL2 protease in cancer cells, E-cadherin. In fact, upon RHBDL2-dependent cleavage of E-cadherin on the cell surface, cancer cell migration increased, likewise in response to E-cadherin knock-down, and independent of the regulation of cell-cell adhesion. This is in line with consistent evidence indicating E-cadherin as a suppressor of cell motility, despite the implicated mechanisms still being debated [7,46,47,48]. For instance, PC3 cells have a mesenchymal phenotype and express E-cadherin that is not implicated in adhesive junctions; yet, gene silencing revealed its role in suppressing cell migration. Moreover, RHBDL2 protease targeted E-cadherin on the cell surface of these cells and promoted cell migration. Thus, our findings implicate RHBDL2 as a novel negative regulator of E-cadherin function in cancer cells.

Intriguingly, upon RHBDL2-dependent cleavage of E-cadherin on the cell surface, we could often detect the soluble E-cadherin extracellular domain in conditioned media; this was observed for diverse tumoral and non-tumoral cell lines, independent from E-cadherin engagement in adherent junctions. When considering that rhomboid proteases target the transmembrane stretch of their targets, it is not surprising that RHBDL2-induced E-cadherin cleavage leads to the shedding of its extracellular domain. If released in sufficient amounts, and surviving further degradation, this protein can accumulate in the conditioned medium and reach the detection threshold. There is evidence that shed E-cadherin has a functional role, but this issue is quite controversial.

In general, the adhesion-independent signaling mechanisms that are mediated by cell surface E-cadherin remain largely unclear. For instance, Chen et al. have previously demonstrated that the intracellular juxtamembrane region of E-cadherin, and not the beta-catenin binding domain, is responsible for suppressing cell migration [46], although the implicated molecular mechanisms have not been sorted out. In contrast, Wong et al. reported that the beta-catenin binding domain of the E-cadherin cytoplasmic tail is required for mediating the suppression of migration/invasion, but this is not dependent on the Tcf1 transcriptional regulator, which suggests the involvement of other still unknown beta-catenin-binding proteins [7]. E-cadherin is also known to interact in cis with other receptors on the cell surface, including integrins [49], and this function could be potentially targeted by the proteolytic activity of RHBDL2. Other studies [39] implicated the extracellular domain of E-cadherin shed in the conditioned medium to act as a trigger for signaling pathways that are potentially capable of promoting cell migration. At the present stage, we are unable to define the motility suppressor mechanism that is mediated by E-cadherin that is disrupted by the cleavage mediated by RHBDL2. In fact, further investigations are needed to clarify the adhesion-independent mechanisms by which E-cadherin suppresses cell migration, and we think that the identification of RHBDL2 as a novel suppressor of E-cadherin function can bring a relevant contribution to this field.

In other experiments, we found that RHBDL2 is further capable of mediating the proteolytic cleavage of the analogous cell surface protein VE-cadherin, but not N-cadherin. VE-cadherin is specifically expressed in endothelial cells, and it is implicated in the maintenance of a compact monolayer and a quiescent non-migratory phenotype. Notably, based on our preliminary evidence, RHBDL2 also induced endothelial migration, a finding which deserves further investigation.

RHBDL2 is well expressed in diverse cancer cell lines; however, we found that its levels are basally low in DU145 prostate cancer cells and endothelial cells. Thus, we used these models to search for extracellular signals eliciting RHBDL2 expression and its activity regulating cadherin function. We found that RHBDL2 is induced by the inflammatory cytokine TNFα, which thereafter leads to a RHBDL2-dependent increase of E-cadherin cleavage. Importantly, while TNFα has been previously reported to induce the expression of members of the metalloprotease superfamily that are also involved in the regulation of cell surface proteins, here we found that TNFα-induced E-cadherin cleavage in tumor cells is reliant on RHBDL2 and independent of MMPs. Furthermore, this finding is of strong potential interest in translational perspective, since RHBDL2 seems to share some relevant substrates with MMPs (including Cadherins and EGF). Notably, cancer therapeutic approaches that are based on MMP-specific inhibitors have failed to achieve significant success [42,50], possibly due to redundant functions that are mediated by RHBDL2 protease induced upon tissue inflammation. Indeed, the loss of function approaches in our experiments indicated that MMPs and RHBDL2 protease are both implicated for E-cadherin targeting in cancer cells. In particular, while our data confirm a relevant role of MMPs for basal E-cadherin shedding, we found that TNFalpha-induced cleavage largely relies on RHBDL2 upregulation. Notably, based on the overexpression experiments, we could demonstrate that the proteolytic cleavage of E-cadherin that was mediated by RHBDL2 is independent of MMP activity.

Fully specific RHBDL2 inhibitor molecules are not currently available; however, the isocoumarin DCI has previously been used for this purpose, and, indeed, we found that it could interfere with RHBDL2-induced E-cadherin cleavage and cancer cell migration, although less efficiently than gene silencing. Our findings open the stimulating prospect that the development of selective RHBDL2 inhibitors will enable their application in cancer therapy regimens, with the aim of hindering cancer cell migration. The idea of targeting RHBDL2 in cancers is particularly intriguing in the face of the above-mentioned correlation between RHBDL2 expression and poor prognosis. Moreover, future preclinical experiments in mouse models will allow for validating the efficacy and feasibility of this therapeutic approach.

## 4. Materials and Methods

### 4.1. Cell Lines

American Type Culture Collection (ATCC, Manassas, VA, USA) provided HEK-293T, MDCK and COS-7 kidney cell lines, PC3 and DU145 human prostate cancer cell lines, MDA-MB468 breast cancer cell line, and human adult aorta endothelial cells immortalized by stably expressing human telomerase catalytic subunit hTERT (Telo-HAEC). All of the tested and authenticated cell lines that were used in this study were cultured in the following media: RPMI-1640 (Sigma-Aldrich, Saint Louis, MO, USA) for PC3, DU145, and MDA-MB468, and DMEM (Sigma-Aldrich) for HEK-293T, MDCK and COS-7 cell lines. The media were supplemented with 10% FBS (Euroclone, Milan, Italy), 2 mM l-glutamine, 100 U/mL penicillin (Sigma-Aldrich), and 100 µg/mL streptomycin (Sigma-Aldrich). The HAEC cells were cultured in EGM-2 Endothelial Cell Growth Medium-2 (Lonza, Basel, Switzerland). Cell lines were incubated in a humidified incubator with 5% CO2 at 37 °C, and they were passaged in culture for fewer than six months after resuscitation. Cell-conditioned media (CM) were collected for analysis after 24 h incubation of cell monolayers with serum-free medium.

### 4.2. Antibodies and Chemicals

Anti-RHBDL2 and anti-E-cadherin antibodies were purchased from Proteintech (Rosemont, IL, USA). Anti-VE-cadherin (clone BV-9) and anti-HA (clone F-7) antibodies were supplied by Santa Cruz Biotechnology. EGFR phosphorylation was detected by a phospho-specific antibody (directed against p-Tyr^1068^) from Abcam (Cambridge, UK). The total and phosphorylated forms of p44/42 MAPK(Erk1/2) and AKT (against pAKT-S^473^ and pMAPK-Thr^202^/Tyr^204^) were detected with antibodies from Cell Signaling Technology (Danvers, MA, USA). Other antibodies that were applied in this study were: for Western Blotting, anti-vinculin (Sigma), anti-N-cadherin (Abcam), anti-Thrombomodulin (D-3, Santa Cruz Biotechnology, Dallas, CA, USA), and anti-RFP (Rockland Immunochemicals Inc., Limerick, PA, USA), for immunofluorescence anti-E-cadherin (clone 36/E, BD Transduction Laboratories) and anti-VE-cadherin (clone F-8, Santa Cruz Biotechnology). The secondary antibodies were purchased from Jackson Laboratories (Bar Harbor, ME, USA). The E-cadherin function-blocking antibody DECMA-1 was purchased from Sigma Aldrich. Santa Cruz Biotechnology supplied the IKK inhibitor IKK-16, while the metalloproteases inhibitors BB94 and Marimastat and the serine protease inhibitor 3,4-dichloroisocoumarin (DCI) were from Sigma. Human recombinant TNFα was purchased from Peprotech (Rocky Hill, NJ, USA).

### 4.3. Cell Proliferation Analysis

The tumor cells were seeded in multiple 96-well plates at an initial density of 1.5– 3 × 10^3^ cells per well (depending on the cell line), and subsequently grown in complete medium. At each experimental time point, one multiwell dish was fixed with 11% glutaraldehyde, stained with crystal violet, and the absorbance was then read while using a standard colorimetric system at 595 nm.

### 4.4. Transwell Migration Assays

Haptotactic migration assays were performed while using Transwell^®^ chamber inserts with a porous polycarbonate membrane (8 μm pore size) (Corning Costar Incorporated, Corning, NY, USA). Briefly, the lower side of the filter was coated with 10 μg/mL fibronectin and blocked with 0.2% BSA. Approximately 5 × 10^4^ cells were added in the upper chamber, and then allowed to migrate through the filter towards the lower chamber. In parallel, the same volume of cell suspension was seeded in cell culture multiwell dishes to check for equal cell loading. When assessing the migration ability in basal conditions, the cells were allowed to migrate for 6 h. In the experiments performed in presence of DCI or Marimastat, cells were pre-treated for 6 h, and then allowed to migrate overnight in presence of the inhibitor. To assess the impact of TNFα, the cells were pre-treated for 6h in order to induce RHBDL2 expression, and then allowed to migrate overnight without further stimulation. At the end of the experiment, the non-migrated cells on the upper side of the filter were removed by a cotton swab, followed by fixing with 11% glutaraldehyde and staining with crystal violet. The microscopic images were then quantified either by cell counting or by converting to a binary image and quantifying the integrated pixel values while using ImageJ (1.47v, National Institutes of Health, Bethesda, MD, USA).

### 4.5. Haptotactic Migration Assays with xCELLigence System

In some experiments, haptotatic migration was monitored in real-time by xCELLigence (Acea Biosciences Inc., San Diego, CA, USA), an electrical impedance-based system in which microelectronic sensor arrays are integrated into the bottom of microplate wells, allowing for cells in the wells to be constantly monitored. The xCELLigence system is based on the Real-Time Cell Analyzer (RTCA) instrument. Its software converts impedance values to obtain parameters, such as cell index (CI), mean values, and standard deviation (SD). Data expressed in CI units can then be exported for any type of mathematical and statistical analyses. For migration assays, the bottom side of the upper chamber (the side facing the lower chamber) of a CIM-Plate 16 was coated with 30 μL of fibronectin (10 μg/mL) for 30 min. inside the tissue culture hood. Each lower chamber well was first filled with 160 μL of serum-free medium (with or without conditioned media from RHBDL2-overexpressing cells) and then assembled to the upper chamber. The assembled plate was incubated at 37 °C for one hour to equilibrate (30 μL of serum-free medium was added to each well of the upper chamber). The PC3 and DU145 cells were resuspended at a final concentration of 30,000 cells/100 μL. The BLANK step was started to measure the background impedance of cell culture medium, which was then used as reference impedance for calculating the CI values. 100 μL of cell suspension (30,000 cells) was then added to each well of the upper chamber. The CIM-Plate 16 was placed in the RTCA DP Instrument that was equilibrated in a CO2 incubator. Cell migration was continuously monitored while using the RTCA DP Instrument.

### 4.6. Wound Healing Migration Assays

Wound healing assays were performed in confluent cell monolayers that were grown in 24-well plates. A pipette tip was used to make one scratch; the cell monolayers were washed twice and images were taken at starting time point, followed by incubation in medium that was supplemented with 5% FBS, in the presence or absence of the E-cadherin function-blocking antibody DECMA-1 (20µg/mL). The wounds were photographed again after 24 h and the images were aligned and analyzed to score for relative wound closure (based on measurement of residual wound area, when compared to the starting point per each well).

### 4.7. Lentiviral-Mediated shRNA or Gene Transfer

For shRNAs, pLKO.1 plasmids containing human RHBDL2-specific (clone ID TRCN0000048601) or E-cadherin specific (clone ID TRCN0000039666) shRNAs were purchased from Sigma. For overexpression experiments, human RHBDL2 ORF was cloned in the lentiviral expression construct pLenti6/V5 from Invitrogen. The non-replicating viral particles containing constructs expressing cDNA or shRNAs (or Empty Vectors, as control) were produced in HEK-293T packaging cells by the calcium phosphate precipitation method, as previously described [51]. Host cells were then incubated with viral particles-containing media, in the presence of polybrene 8 µg/mL, for 12 h. This method ensured a stable gene transfer with very high efficiency, without the need to select individual cell clones. In addition, to rule out the variability of biological responses, at least three independent gene-transduced cell batches were analyzed.

### 4.8. RNA Isolation and Real-Time Quantitative PCR

RNeasy Protect Mini Kit (Qiagen, Venlo, The Netherlands) was used to isolate total RNA from tumor cell lines or tissues, according to the manufacturer’s instructions. cDNA was synthesized according to standard procedures, while using M-MLV Reverse Transcriptase (Promega, Madison, WI, USA) and random primers (Promega). Gene expression was analyzed using the following specific primer pairs by applying Sybr-green technology: RHBDL2 (AGAGAGGATGGGGGAGGTAA, AAGATGCCTGTGTCCAACG), E-cadherin (GGCAGTGTCTCTCCAAATCC, GGTCTCTCTCACCACCTCCA) GAPDH (GAAGGTGAAGGTCGGAGTC, GAAGATGGTGATGGGATTTC), and β-actin (CACTCTTCCAGCCTTCCTTC, GCTGTGCTACGTCGCCCTG). Real-time PCR analysis was performed while using the Applied Biosystems 7900HT Fast Real-time PCR System (Applied Biosystems, Foster City, CA, USA).

### 4.9. Transient Transfection

The expression constructs for mouse RHBDL2 (tagged with a triple N-terminal HA-tag), and its catalytically inactive S186A derivative, were kindly provided by Matthew Freeman [23]. The expression construct for VE-cadherin (#54959 from Addgene, Watertown, MA, USA) was kindly provided by Prof. Guido Serini. COS-7 cells were transiently transfected while using the DEAE-Dextran method, HEK-293T by the calcium phosphate precipitation method.

### 4.10. Immunoprecipitation and Western Blotting Analysis

For Immunoprecipitation experiments, the transiently transfected HEK-293T cells were lysed in an ice cold lysis buffer (EB) containing 1% Triton X-100, 10 mM Tris, pH 7.6, 50 mM NaCl, 0.1% BSA, and phosphatases and proteases inhibitors: 1 mM PMSF, 1 mM Na3VO4, 1 mM NaF, 1 mM aprotinin, 1 mM leupeptin, and 1 mM pepstatin. Equal amounts of cell lysates were incubated with Protein A-Sepharose beads that were coated with specific antibodies. After 2 h of incubation at 4 °C, the beads were extensively washed, and the immunoprecipitated proteins were denaturated by boiling in Laemmli buffer. The total protein extracts were directly obtained by cell lysis in LB-SDS buffer (25% Tris HCl 0.5 M pH 6.8, 25% SDS 10%, 50% ddH_2_0); the total protein content was measured and an equal amount was loaded for analysis by western blotting. For the collection of conditioned media (CM), cells in diverse conditions were previously seeded in equal number and then incubated for 24 h in the same volume of serum-free culture medium. CM was then filtered to remove the cell debris, and an equal volume from each sample was subjected to protein analysis. Western blotting was performed according to standard protocols. Signal detection was completed by enhanced chemioluminescence (ECL, Amersham Biosciences, GE Healthcare, Chicago, IL, USA).

### 4.11. Immunofluorescence

For immunofluorescence analysis, cells that were grown on round coverslips were fixed in 4% paraformaldehyde for 15 min., permeabilized with 0.1% Triton/phosphate-buffered saline (PBS) for 3 min. at room temperature, and then blocked by 5% donkey serum for 30 min. In alternative, for experiments in non-permeabilizing conditions, the cells were treated with Zinc Fixative (6.05g Tris, 0.35g Ca(C_2_H_3_O_2_)_2_, 2.5g Zn(C_2_H_3_O_2_)_2_, 2.5g ZnCl_2_, 3.8 mL HCl 37%) for 10 min. at room temperature. The fixed cells were then incubated with primary antibodies for 1 h at room temperature, followed by incubation with the fluorochrome-conjugated secondary antibodies for 30 min. at room temperature. F-actin was stained by using fluorescent-Phalloidin conjugates. Nuclei were stained with 4,6-diamidino-2-phenylindole (DAPI). The coverslips were then washed and mounted on slides. The images were acquired with a confocal laser-scanning microscope (SPEII DM5500 CSQ; Leica Microsystems CMS GmbH, Wetzlar, Germany) that was equipped with a 63×/1.30 HCX Plan-Apochromat oil immersion objective lens (ACS APO 63×/1.30 oil CS 0.17/E, 0.16) while using Leica LAS AF software version 2.6.3.8173.

### 4.12. Statistical Analysis

Statistical significance was assessed by two tailed Student’s *t*-test, calculated with GraphPad prism software (version 8.0.0, GraphPad Software, Inc., San Diego, CA, USA). The error bars represent the SD, as indicated in each figure legend. All of the experiments were repeated at least three times (biological replicates) with consistent results, though in some cases figures show one representative experiment. Statistical significance is indicated by asterisks in the figures, as follows: * *p* < 0.05; ** *p* < 0.005; *** *p* < 0.0005.

## Supplementary Materials

Supplementary materials can be found at https://www.mdpi.com/1422-0067/20/23/5958/s1.

## Abbreviations

RHBDL2	Rhomboid-like-2
MMP	Matrix Metalloprotease
TNFα	Tumor Necrosis Factor α
EGF	Epidermal Growth Factor
ADAM	A Disintegrin And Metalloproteinase
