# Supplementary material for: Rhomboid-Like-2 Intramembrane Protease Mediates Metalloprotease-Independent Regulation of Cadherins
Source: Int J Mol Sci. 2019 Nov 27;20(23):5958. doi: 10.3390/ijms20235958 (PMC6928865; doi:10.3390/ijms20235958)
Supplement: Supplementary file 1 [file ijms-20-05958-s001.pdf]

SUPPLEMENTAL FIGURES and Legends

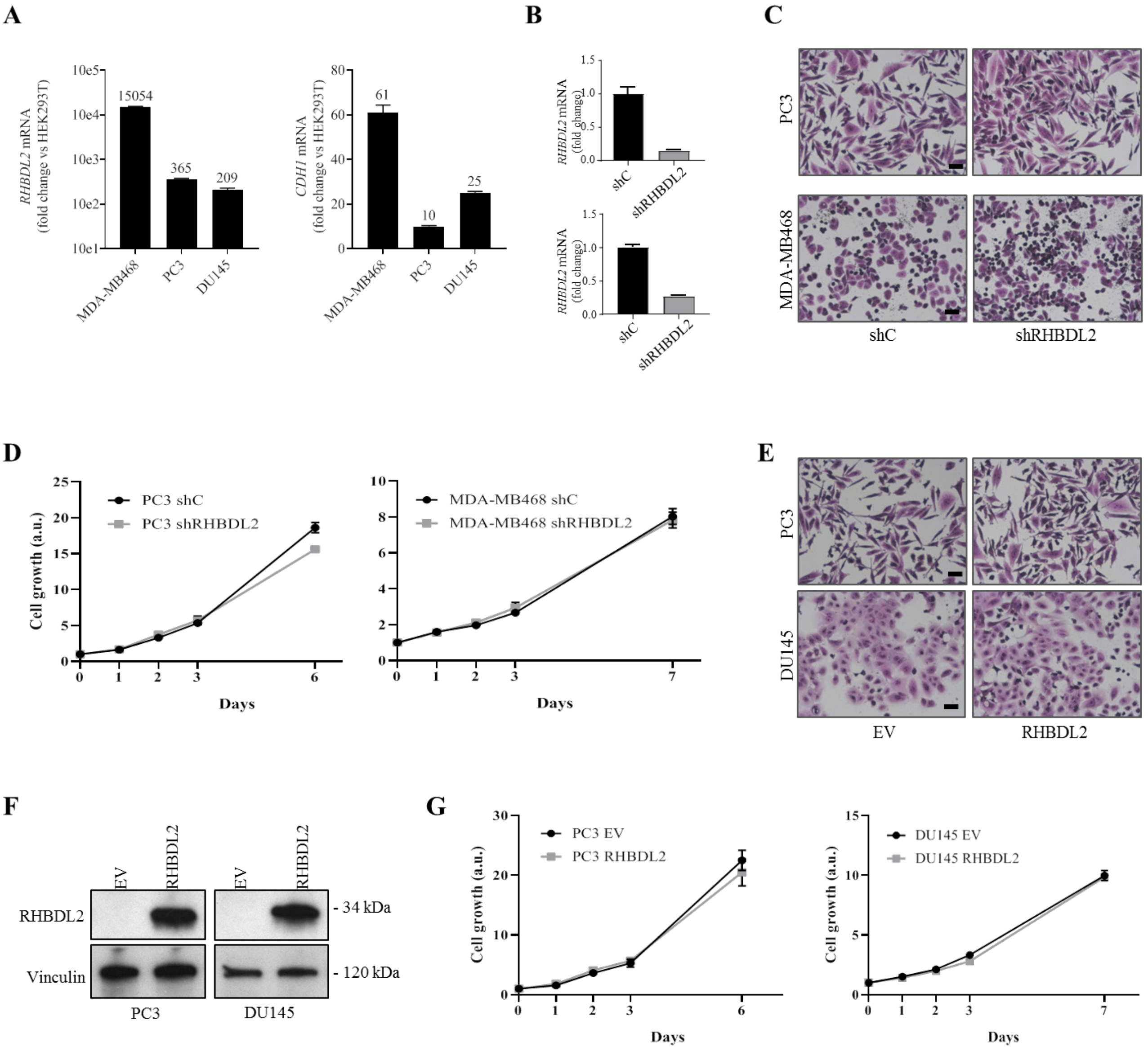

FIGURE S1. (**A**) qPCR analysis showing the relative expression of RHBDL2 and E-cadherin in MDA-MB468, PC3 and DU145, compared to HEK293T cells (set to 1). (**B**) RHBDL2 expression analysis by qPCR in PC3 (upper row) and MDA-MB468 (lower row) carcinoma cells, either control (shC) or silenced for RHBDL2 (shRHBDL2); cells were fixed and stained with crystal violet, scale bars: 100 μm. (**C**) Microscopic images of the same cells analyzed in panel (B). (**D**) Growth curve of the same PC3 (left) and MDA-MB468 (right) cells, control and silenced for RHBDL2, shown in previous panels; three independent experiments were performed. (**E**) Microscopic images of PC3 (upper row) and DU145 (lower row) prostate carcinoma cells, stably transduced with a RHBDL2-expressing construct or an empty vector (EV); cells were fixed and stained with crystal violet, scale bars: 100 μm. (**F**) Western blotting analysis to validate RHBDL2 overexpression in the cells shown in panel (E); vinculin provided a protein loading control. (**G**) Growth curve of PC3 (left) and DU145 (right) cells, control (EV) and stably overexpressing RHBDL2; three independent experiments were performed.

 Int. J. Mol. Sci. **2019**, *20*, 5958; doi:10.3390/ijms20235958

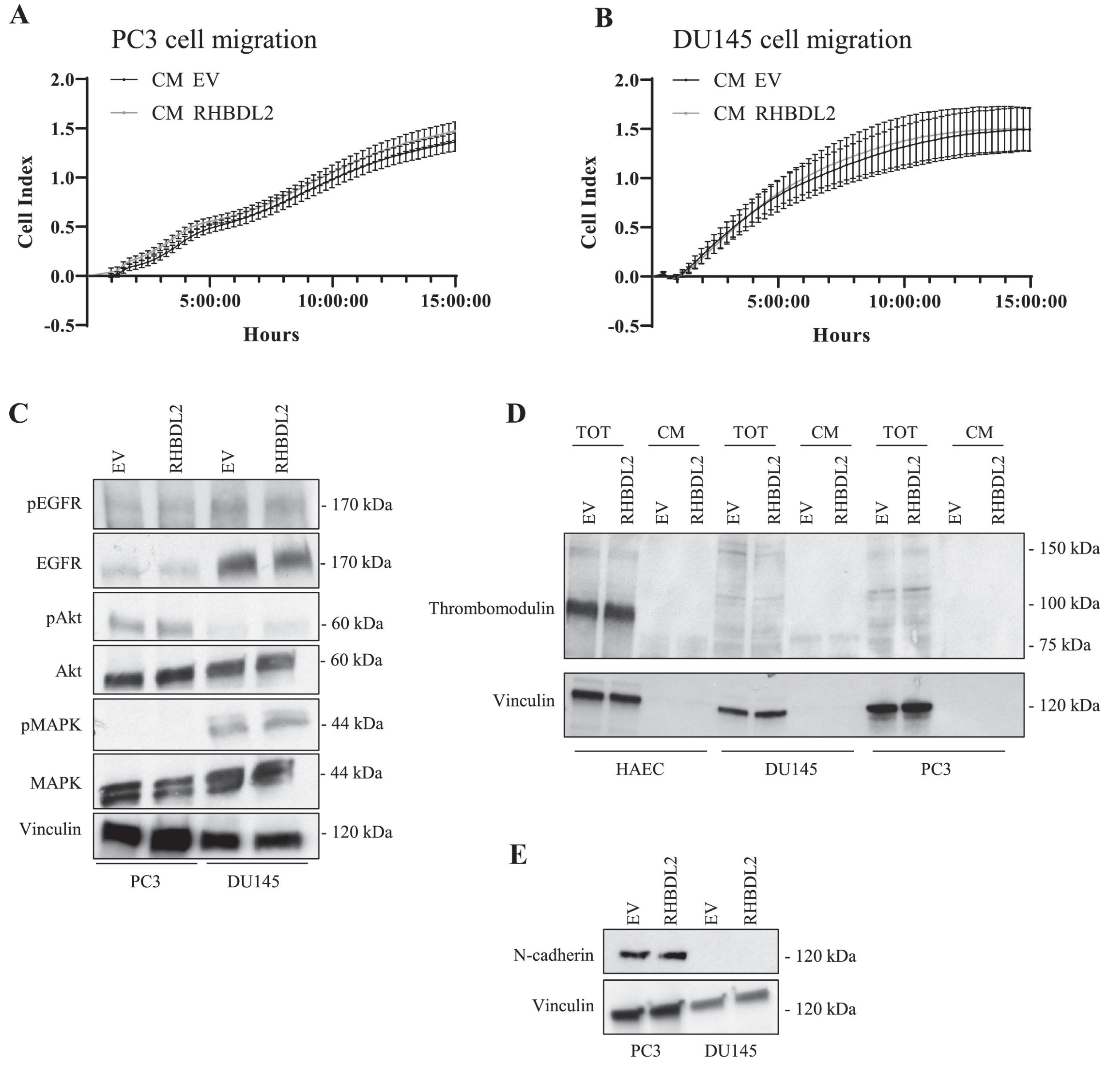

FIGURE S2. (**A**, **B**) Migration of PC3 (A) and DU145 (B) cells, monitored in real time as Cell Index (by xCELLigence device), in presence of conditioned media (CM) from DU145 control (EV) or stably overexpressing RHBDL2 cells. (**C**) Western Blotting analysis of EGFR, Akt and p44/42 MAPK (Erk1/2), total and phosphorylated proteins, in PC3 and DU145 cells subjected to stable RHBDL2 overexpression (or transduced with an empty vector, EV); vinculin staining provided a loading control. (**D**) Western Blotting analysis of Thrombomodulin in total lysates (TOT) and conditioned media (CM) of HAEC, DU145 and PC3 cells, stably transduced with a RHBDL2-expressing construct or an empty vector (EV). HAEC cells were used as a positive control for Thrombomodulin expression (in qPCR analysis, delta Ct of Thrombomodulin vs Actin were the following: HAEC: 7; DU145: 12; PC3: 13.5). (**E**) Western Blotting analysis of N-cadherin expression in PC3 and DU145 cells, either control (EV) or upon RHBDL2 overexpression. N-cadherin was below detection threshold in DU145 cells. Vinculin staining provided a loading control.

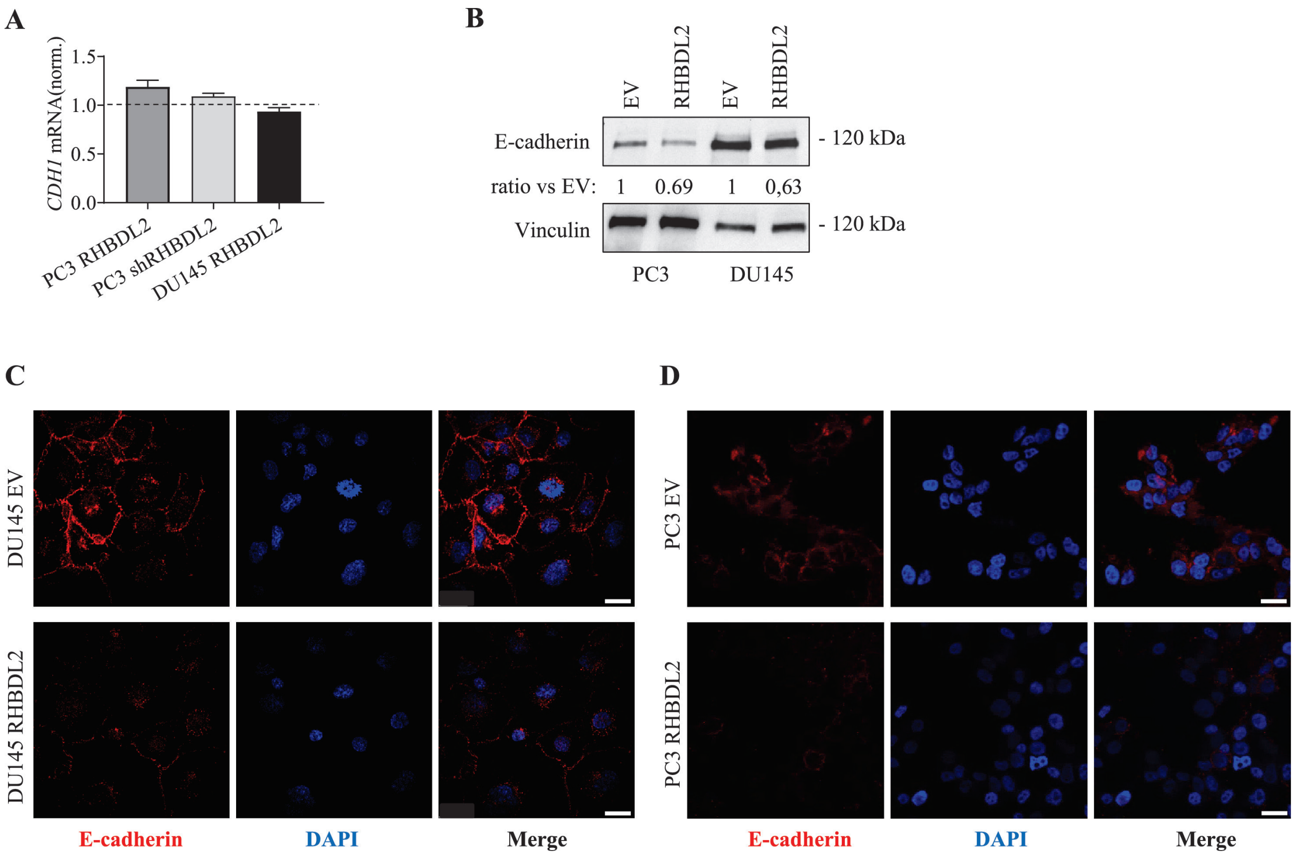

FIGURE S3. (**A**) qPCR analysis of *CDH1* mRNA expression variations in the same PC3 or DU145 cells subjected to RHBDL2 genetic manipulation analyzed in main Fig. 2. In the plot, expression levels were normalized to those found in respective control cells, set as 1 (dashed line). (**B**) Western Blotting analysis of E-cadherin expression in PC3 and DU145 cells, either control (EV) or upon RHBDL2 overexpression. E-cadherin protein levels are significantly lower in PC3 than in DU145 cells. Vinculin staining provided a loading control. (**C, D**) Immunostaining of E-cadherin extracellular domain exposed on the cell surface in non-permeabilized DU145 (**C**) and PC3 (**D**) cells, transduced with a RHBDL2-expressing construct or an empty vector (EV); the nuclei were eventually stained by DAPI (blue). Scale bars: 25 μm.

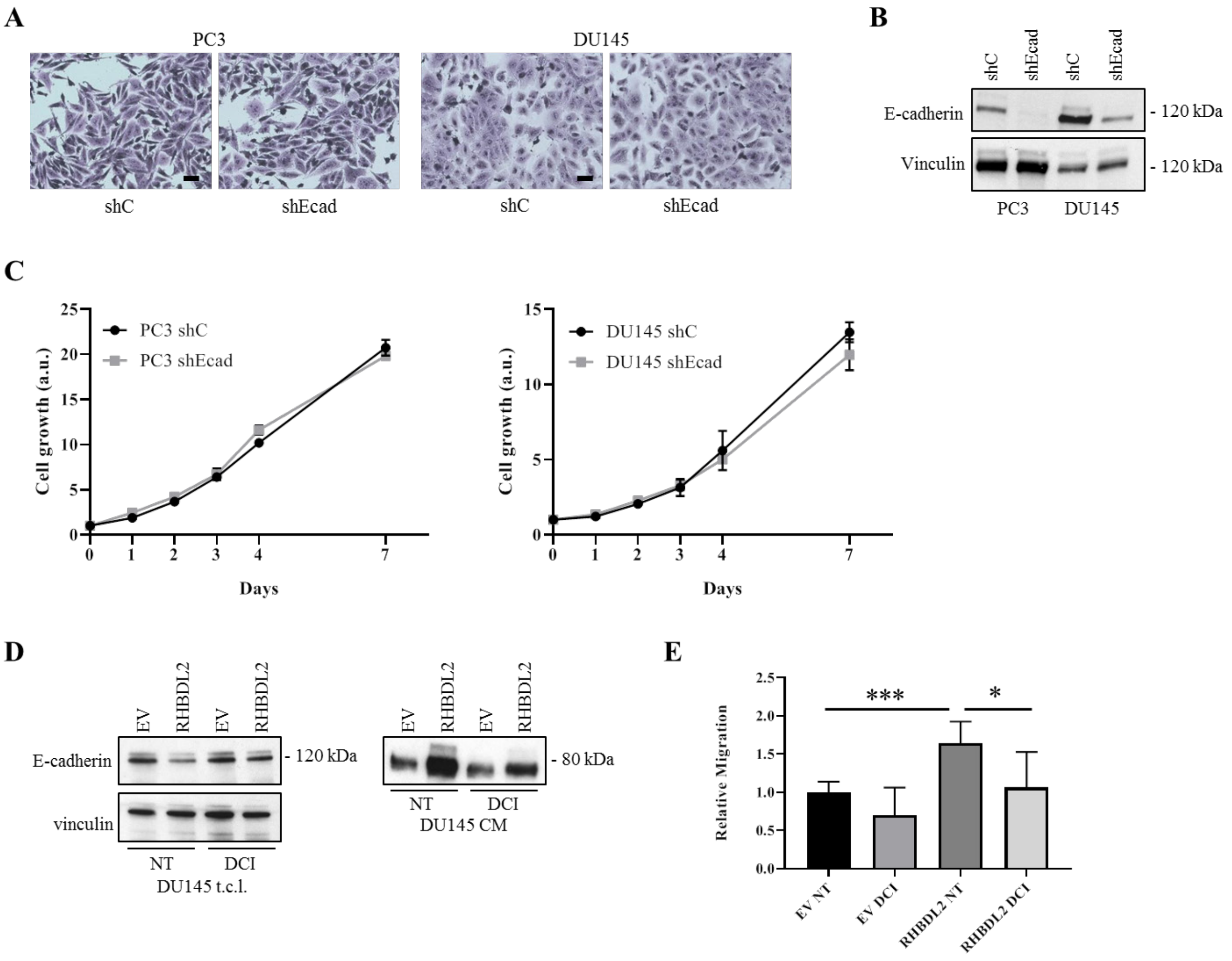

FIGURE S4. (**A**) Microscopic images of PC3 (left) and DU145 (right) prostate cancer cells, stably transduced with scramble shRNA (shC) or a shRNA specifically targeting E-cadherin (shEcad); cells were fixed and stained with crystal violet, scale bars: 100 μm. (**B**) Western blotting analysis to validate E-cadherin silencing in the cells shown in panel (A); vinculin provided a protein loading control. (**C**) Growth curve of PC3 (left) and DU145 (right) cells, either control (shC) or stably silenced for E-cadherin (shEcad); three independent experiments were performed. (**D**) Western Blotting analysis of E-cadherin expression in total lysates and conditioned media (CM) of DU145 cells transduced with RHBDL2 or an empty vector (EV), and maintained in the presence (or absence) of the RHBDL2-inhibitor DCI (5 μM). Vinculin staining provided a loading control. CM was collected after 24 h of serum starvation. (**E**) The migration of DU145 cells, either control (EV) or stably overexpressing RHBDL2, was assessed using Transwell chamber inserts, in the presence (or absence, NT) of 5 μM DCI. Data are the mean ± SD from three independent experiments. Statistical significance: * $p < 0.05$; *** $p < 0.0005$.

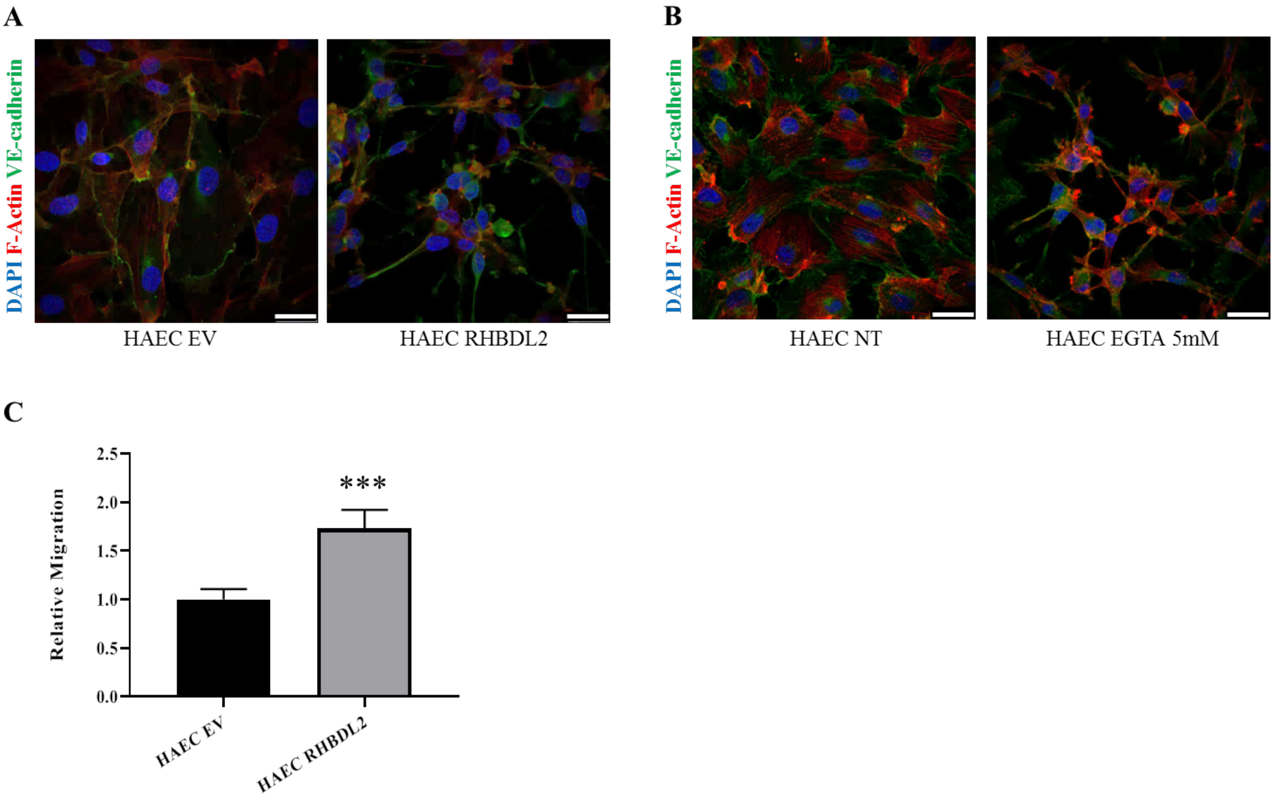

FIGURE S5. (**A**) Immunofluorescence analysis of VE-cadherin (green) in HAEC cells stably transduced with a RHBDL2-expressing construct, or an empty vector (EV). F-actin was stained by fluorescent Phalloidin (red) and nuclei by DAPI (blue). Scale bars: 25 µm. (**B**) Immunofluorescence analysis of VE-cadherin (green) in HAEC cells kept for 30 min in normal medium (NT) or in Ca^{++}-depleted conditions, obtained by adding EGTA 5 mM to the medium. F-actin was stained by fluorescent Phalloidin (red) and nuclei by DAPI (blue). Scale bars: 25 µm. (**C**) Quantification of the migratory ability of HAEC cells control (EV) or stably overexpressing RHBDL2, assessed using transwell chamber inserts. Data are the mean ± SD from three independent experiments. Statistical significance: *** $p < 0.0005$.

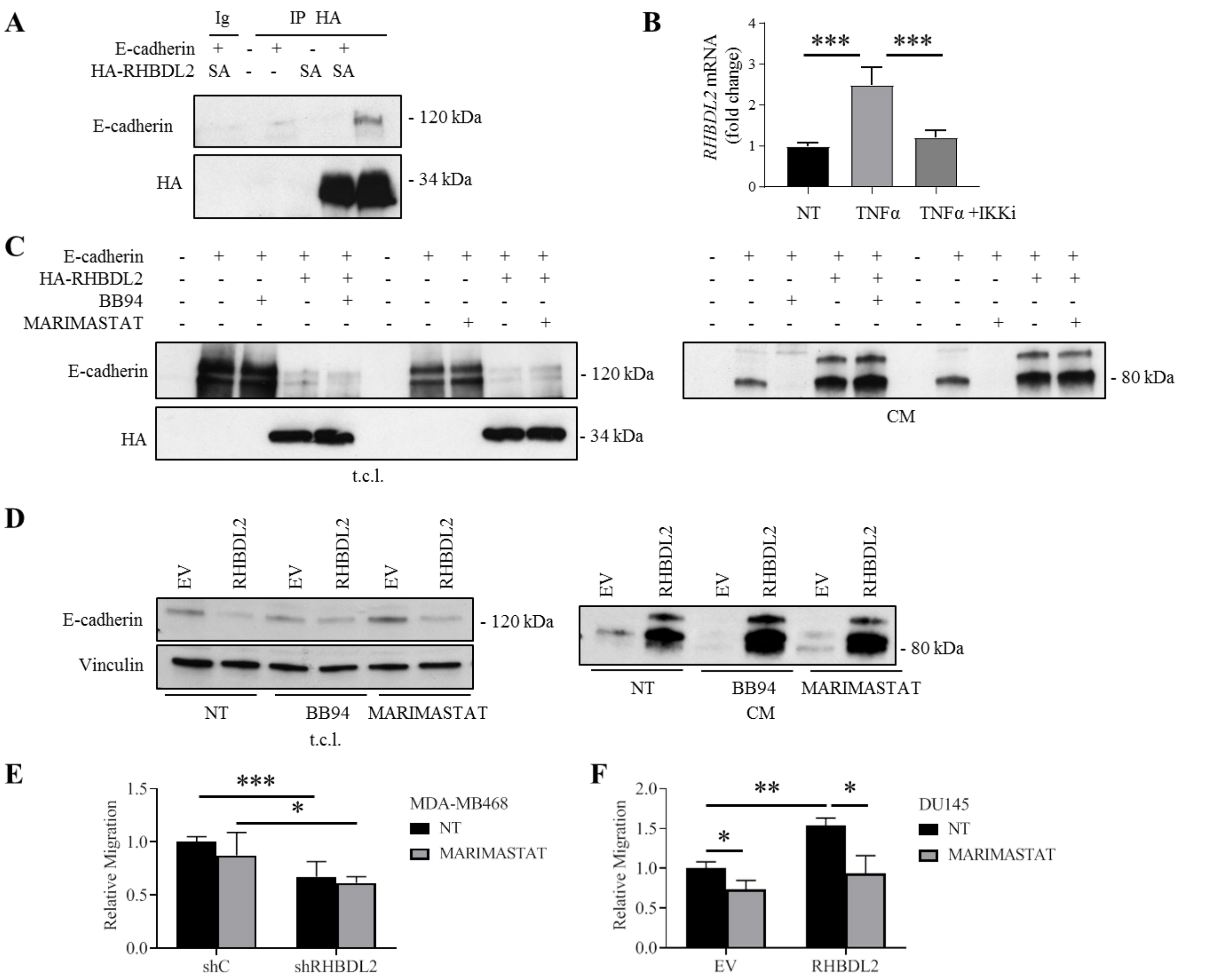

FIGURE S6. (**A**) Lysates from HEK293T cells transfected with E-cadherin and/or with HA-tagged RHBDL2-SA were immunoprecipitated with anti-HA or an unrelated antibody (Ig). Western Blotting against E-cadherin revealed its co-immunoprecipitation with RHBDL2. (**B**) NF-kB pathway inhibition impairs TNFα induction of RHBDL2. DU145 were treated with TNFα 10 ng/ml alone for 6 h, or pre-treated for 2 h with NF-kB inhibitor IKK-16 (1 μM) before addiction of TNFα, and mRNA levels of RHBDL2 were analyzed by qPCR. Data are the mean ± SD from three independent experiments. Statistical significance: ***, $p < 0.0005$. (**C**) Western Blotting showing total lysates (TOT) and conditioned media (CM) of HEK-293T cells transfected with E-cadherin and HA-tagged RHBDL2, in presence or absence of MMP inhibitors BB94 (20 μM) or Marimastat (50 μM). Expression of HA-tagged rhomboid is shown below. CM was collected after 24 h of serum starvation. (**D**) Western Blotting showing total lysates (TOT) and conditioned media (CM) of DU145 cells stably transduced with a RHBDL2-expressing construct or an empty vector (EV), in presence or absence of MMP inhibitors BB94 (20 μM) or Marimastat (50 μM). Vinculin staining was used as a loading control. CM was collected after 24 h of serum starvation. The migration of MDA-MB468 cells (**E**), either control (shC) or silenced for RHBDL2 (shRBDL2), and of DU145 cells (**F**), either control (EV) or overexpressing RHBDL2, was assessed using Transwell chamber inserts, in the presence (or absence, NT) of Marimastat 50 μM. Data are the mean ± SD from three independent experiments. Statistical significance: * $p < 0.05$, ** $p < 0.005$; *** $p < 0.0005$.

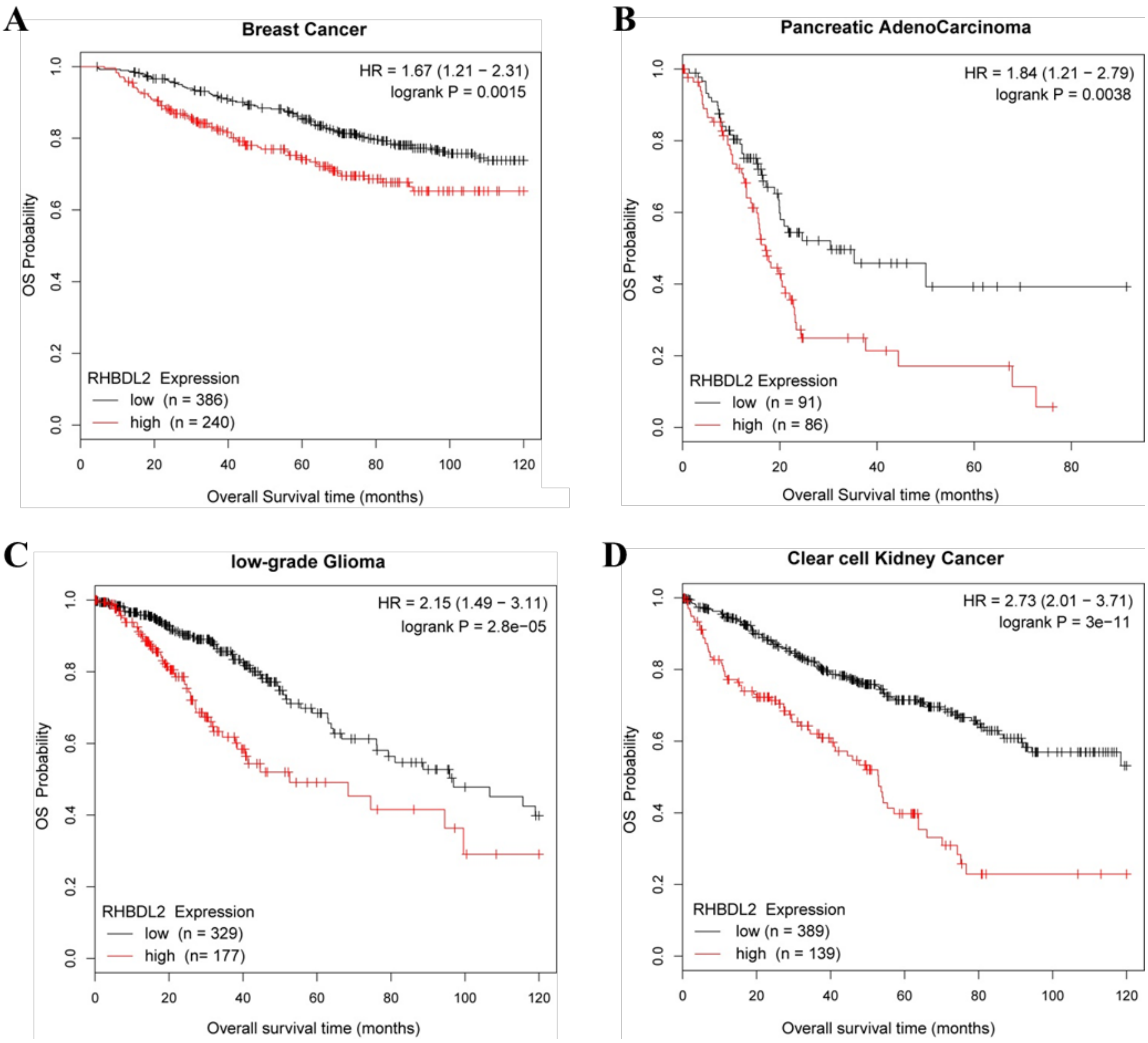

FIGURE S7. Correlation of patient overall survival with RHBDL2 expression in the human cancers. (**A-D**) Kaplan-Meier analysis (generated with http://kmplot.com analysis platform) of the Overall Survival (OS) of the indicated cancer patients, stratified as expressing high or low levels of RHBDL2 mRNA in tumor samples. Indicated in the graphs are Hazard Ratio (HR) and statistical significance (logrank P value) per each tumor type, i.e. breast carcinoma (A), pancreatic adenocarcinoma (B), low-grade glioma (C), and clear cell kidney cancer (D).
